# Psychosis Beyond the Seizure: A Neuropsychiatric Case Study in Postoperative Temporal Lobe Epilepsy

**DOI:** 10.1192/j.eurpsy.2026.11537

**Published:** 2026-07-31

**Authors:** F. Bayam, R. Alves, M. Menino, S. Marques, S. Meleiro, I. Marques, J. Melim, C. Almeida, C. Bayam, C. Laureano

**Affiliations:** 1 Serviço de Psiquiatria e Saúde Mental, Unidade Local de Saúde da Região de Leiria, Leiria; 2 Pedopsiquiatria, Unidade Local de Saúde de Coimbra, Coimbra, Portugal

## Abstract

**Introduction:**

The overlap of epilepsy and psychiatric pathology presents a diagnostic challenge, particularly in patients with structural brain lesions. Epilepsy increases the risk of psychosis, classically defined as ictal, peri-ictal, or interictal. In frontotemporal disease, psychopathology may also arise from network dysfunction, encephalopathy, or neurodegeneration. Distinguishing among these processes is essential for management and prognosis.

**Objectives:**

To illustrate the complexity of psychosis and cognitive dysfunction in structurally mediated epilepsy and emphasize the value of an integrative, multidisciplinary approach.

**Methods:**

A single case was examined through longitudinal neuropsychiatric assessment, neuroimaging, cerebrospinal fluid (CSF) analysis, electroencephalography (EEG), and neuropsychological testing, with coordinated psychiatric–neurological management.

**Results:**

A 56-year-old female underwent right frontotemporal meningioma resection (2019) and subsequently developed focal epilepsy controlled with levetiracetam. With no prior psychiatric history, she presented in 2021 with acute psychosis amid psychosocial stressors, requiring psychiatric admission and achieving remission under antidepressant and low-dose antipsychotic therapy. In 2025, she re-presented after a near-lethal suicide attempt, with persecutory delusions, disorganization, irritability, insomnia, episodic incontinence, fluctuating attention, and partial amnesia. Levetiracetam was tapered and replaced with eslicarbazepine. EEG demonstrated diffuse slowing without epileptiform discharges. MRI showed stable postoperative changes without recurrence or new lesions. CSF, including autoimmune and viral panels, was negative. Neuropsychological testing revealed mild impairment, with deficits in executive function and verbal memory, consistent with frontotemporal dysfunction. The fluctuating course suggested interictal psychosis complicated by behavioral encephalopathy or evolving neurodegenerative change.

**Conclusions:**

This case highlights the diagnostic ambiguity at the neuropsychiatric interface in structural epilepsy, where psychosis, cognitive decline, and neurological signs coexist. Absence of epileptiform discharges does not exclude seizure-related psychopathology, given the limited sensitivity of routine EEG for mesial temporal or deep cortical foci. Structural frontotemporal lesions may predispose to both epileptiform activity and psychiatric dysregulation via network disruption. Management requires moving beyond rigid psychiatric–neurological distinctions toward integrated multidisciplinary frameworks. Longitudinal surveillance, advanced electrophysiological monitoring, and individualized treatment are essential for accurate diagnosis and prevention of recurrence.

**Disclosure of Interest:**

None Declared

